# Biochemical Alterations during the Obese-Aging Process in Female and Male Monosodium Glutamate (MSG)-Treated Mice

**DOI:** 10.3390/ijms150711473

**Published:** 2014-06-27

**Authors:** René J. Hernández-Bautista, Francisco J. Alarcón-Aguilar, María Del C. Escobar-Villanueva, Julio C. Almanza-Pérez, Héctor Merino-Aguilar, Mina Konigsberg Fainstein, Norma E. López-Diazguerrero

**Affiliations:** Post-Grade in Experimental Biology, Division of Health and Biological Sciences, Metropolitan Autonomous University, A.P. 55-535, D.F. Mexico, Mexico; E-Mail: rjhb11@yahoo.com.mx; Laboratory of Pharmacology, Department of Health Sciences, Division of Health and Biological Sciences, Metropolitan Autonomous University, A.P. 55-535, D.F. Mexico, Mexico; E-Mails: aaaf@xanum.uam.mx (F.J.A.-A.); mcev@xanum.uam.mx (M.D.C.E.-V.); jcap@xanum.uam.mx (J.C.A.-P.); hmerino@ciencias.unam.mx (H.M.-A.); Laboratory of Bioenergetics and Cellular Aging, Department of Health Sciences, Division of Health and Biological Sciences, Metropolitan Autonomous University, A.P. 55-535, D.F. Mexico, Mexico; E-Mail: mkf@xanum.uam.mx

**Keywords:** obesity, aging, monosodium glutamate, diabetes, insulin resistance, inflammation, cytokines

## Abstract

Obesity, from children to the elderly, has increased in the world at an alarming rate over the past three decades, implying long-term detrimental consequences for individual’s health. Obesity and aging are known to be risk factors for metabolic disorder development, insulin resistance and inflammation, but their relationship is not fully understood. Prevention and appropriate therapies for metabolic disorders and physical disabilities in older adults have become a major public health challenge. Hence, the aim of this study was to evaluate inflammation markers, biochemical parameters and glucose homeostasis during the obese-aging process, to understand the relationship between obesity and health span during the lifetime. In order to do this, the monosodium glutamate (MSG) obesity mice model was used, and data were evaluated at 4, 8, 12, 16 and 20 months in both female and male mice. Our results showed that obesity was a major factor contributing to premature alterations in MSG-treated mice metabolism; however, at older ages, obesity effects were attenuated and MSG-mice became more similar to normal mice. At a younger age (four months old), the Lee index, triglycerides, total cholesterol, TNF-α and transaminases levels increased; while adiponectin decreased and glucose tolerance and insulin sensitivity levels were remarkably altered. However, from 16 months old-on, the Lee index and TNF-α levels diminished significantly, while adiponectin increased, and glucose and insulin homeostasis was recovered. In summary, MSG-treated obese mice showed metabolic changes and differential susceptibility by gender throughout life and during the aging process. Understanding metabolic differences between genders during the lifespan will allow the discovery of specific preventive treatment strategies for chronic diseases and functional decline.

## 1. Introduction

Obesity has generally been considered an epidemic related to lifestyle, which not only occurs in the young and adult population, but is also observed in elderly people. Obesity is a health problem defined as an abnormal or excessive fat accumulation [1], due to an imbalance in energetic metabolism homeostasis, generated by multiple genetic and environmental factors, usually controlled by the central nervous system [2]. According to the World Health Organization, over 300 million obese adults and 42 million overweight children undergo this condition [3].

Aging has been defined as the molecular, biochemical and cellular progressive decline during the lifespan. Aging deterioration may depend on the interplay between intrinsic and extrinsic factors [4], as well as on the organism’s capability to respond to different stressors, in order to counteract their effects or adapt to the new conditions.

Both obesity and aging have been defined as low-grade systemic inflammation processes and represent risk factors for a wide range of diseases, including insulin resistance (IR) [5], type 2 diabetes, dyslipidemia and cardiovascular disease [2,6,7]. In obesity, the intra-abdominal adipose tissue growth promotes increased pro-inflammatory cytokines infiltration and activation, such as tumor necrosis factor (TNF-α) and interleukin 6 (IL-6) [8], which denotes the primary causes for chronic inflammation, morbidity and mortality risk. Nevertheless when it comes to obesity, it is important to consider the significant differences associated with gender, which are mostly related to adipose tissue distribution and inflammation. In this regard, previous studies found that female mice accumulate subcutaneous body fat, whereas fat in male mice is stored in the visceral region [7].

Conversely, during the aging process, chronic low-grade systemic inflammation, poor physical performance and altered energetic metabolism, combined with obesity, potentiate the risk of developing the cited diseases. The visceral adiposity increment along with the accumulation of senescent cells, which are characterized by an inflammatory phenotype, lead to a pro-inflammatory cytokine increase in plasma, which, in turn, interferes with insulin signaling. This low-grade systemic inflammation, termed “inflammaging”, is associated with diseases, lipo-toxicity and reduced longevity, and therefore, TNF-α and IL-6 have become frailty markers in humans [9].

Many studies have dealt with obesity’s physiological effects on the organism; however, the potential consequences of obesity during the aging process have not been fully understood. Although some medical complications of obesity in the elderly have been described (metabolic disorders, glucose intolerance, hypertension, dyslipidemia, cardiovascular disease), there are no longitudinal studies that analyze the obesity-aging process during the health span and lifespan.

The obesity model used in this study was generated by monosodium glutamate (MSG) neonatal neuro-intoxication, which has been reported to induce a hypothalamic lesion in the arcuate nucleus and neuro-endocrine alterations in insulin and leptin signaling, among other effects [5,6]. MSG-treated animals develop obesity, which becomes apparent at eight weeks of age [6,10,11], and therefore, it has been acknowledged as a suitable model to study metabolic dysfunction [10,12,13,14].

The aim of this study was to evaluate inflammation markers, biochemical parameters and glucose homeostasis during the lifetime in MSG-treated female and male mice, in order to determine the associated effect and influence of obesity during the aging process.

## 2. Results

### 2.1. Weight, Size and Lee Index Time Courses

**Figure 1:**
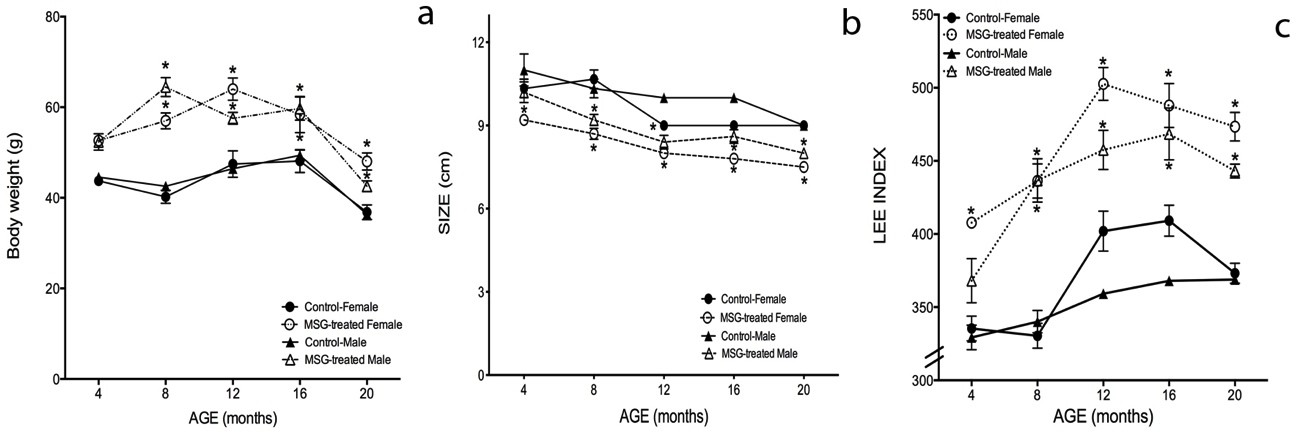
Weight, size and Lee index time courses in male and female MSG-treated mice. Temporal courses of (**a**) body weight; (**b**) size and (**c**) the Lee index were performed, as described in the Materials and Methods section. Measurements were determined at 4, 8, 12, 16 and 20 months of age. Plotted values are the mean ± SE of five mice per group. * *p* < 0.05 *vs.* control; ●, control female; ○, MSG-treated female; ▲, control male; ∆, MSG-treated male.

Gradual increments of body weight over time were observed in obese, as well as in control mice while they aged; however, BW was significantly higher in MSG-treated mice than in their control littermates. BW increased 20%, 42%, 36% and 20% at 4, 8, 12 and 16 months of age in obese female mice, respectively, and 18%, 51%, 23% and 21% in obese male mice (Figure 1a). Interestingly, at 16 months-old and more evident at 20 months-old, BW decreased in all four groups. For MSG female mice, the BW decrease was 33%, and 17% in MSG male mice compared to their controls. At eight months of age, a significant difference between genders was observed, since obese male mice had a higher BW (13%) compared to obese female mice; however, at 12 months-old, this behavior changed, and the MSG females showed a higher BW than the MSG males (Figure 1a) (*p* < 0.05).

The naso-anal length values quantified to determine mice size also lessened in obese and control groups over time. Still, the MSG-treated mice shortened more than the control animals: 14% obese female and 12% obese male mice (Figure 1b). The Lee index (LI) increased in all of the groups during the lifetime, and as expected, the MSG-treated groups obtained significantly higher LI values than their controls. LI values in obese female mice were 21%, 32%, 24%, 19% and 26% higher than control females’ at 4, 8, 12, 16 and 20 months, while obese male mice’s LI values were 11%, 28%, 27%, 27% and 20% superior to their controls, at the same time points measured. In regard to the gender, female mice showed approximately 10% higher LI values than males in both cases (control and obese) (Figure 1c) (*p* < 0.05).

### 2.2. Oral Glucose Tolerance Test (OGTT)

**Figure 2:**
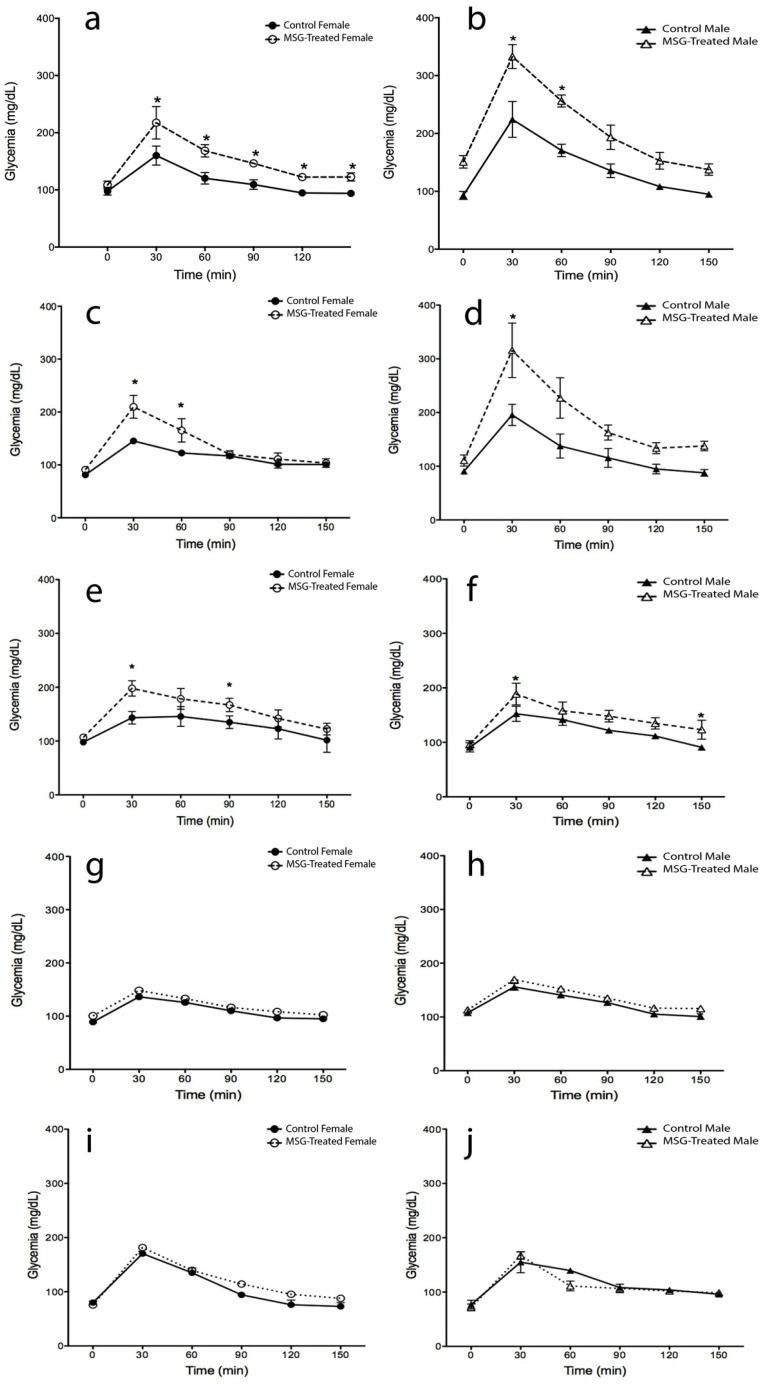
Oral glucose tolerance test (OGTT) in male and female MSG-treated mice. The test was performed by oral administration of dextrose (2 g/kg body wt). Plasma glucose was monitored before (0 min) and 30, 60, 90, 120 and 150 min after dextrose-administration, as described in the Materials and Methods section. Determinations were measured at 4 (**a**,**b**), 8 (**c**,**d**), 12 (**e**,**f**), 16 (**g**,**h**) and 20 (**i**,**j**) months of age. Plotted values are the mean ± SE for five mice per group. * *p* < 0.05 *vs.* control female and male; ●, control female; ○, MSG-treated female; ▲, control male; ∆, MSG-treated male.

Figure 2 shows oral glucose tolerance test (OGTT) alterations during the lifetime in obese and control mice at 4, 8, 12, 16 and 20 months-old. Both female groups (MSG and control) showed a decrease in glucose homeostasis over time, which was only different at the beginning of the study. At four months, 35% higher glycemic values were obtained in MSG-treated mice 30 min after dextrose administration when compared to the control group. This value increased to 44% at 8 months-old (Figure 2a); however, from 12 to 20 months, the differences between groups decreased (Figure 2c,e,g,i) (*p* < 0.05). 

In regards to the male groups, a significant difference was found at 4 and 8 months-old between control and obese mice (48% and 61% higher in MSG mice after 30 min dextrose administration), but from 16 months-on, no significant differences were observed, since MSG-treated mice glycaemia decreased nearly to control values (Figure 2b,d,f,h,j) (*p* < 0.05). 

Notably, males showed higher glucose intolerance levels than females. At 4 months-old, MSG-treated male mice displayed a greater glucose intolerance level of 53% and 52%, respectively at 30 and 60 min, and at 8 months-old, the differences increased to 117% and 85%, respectively. No differences were found in the following points of the OGTT curve, as well as at 12, 16 and 20 months-old.

**Figure 3:**
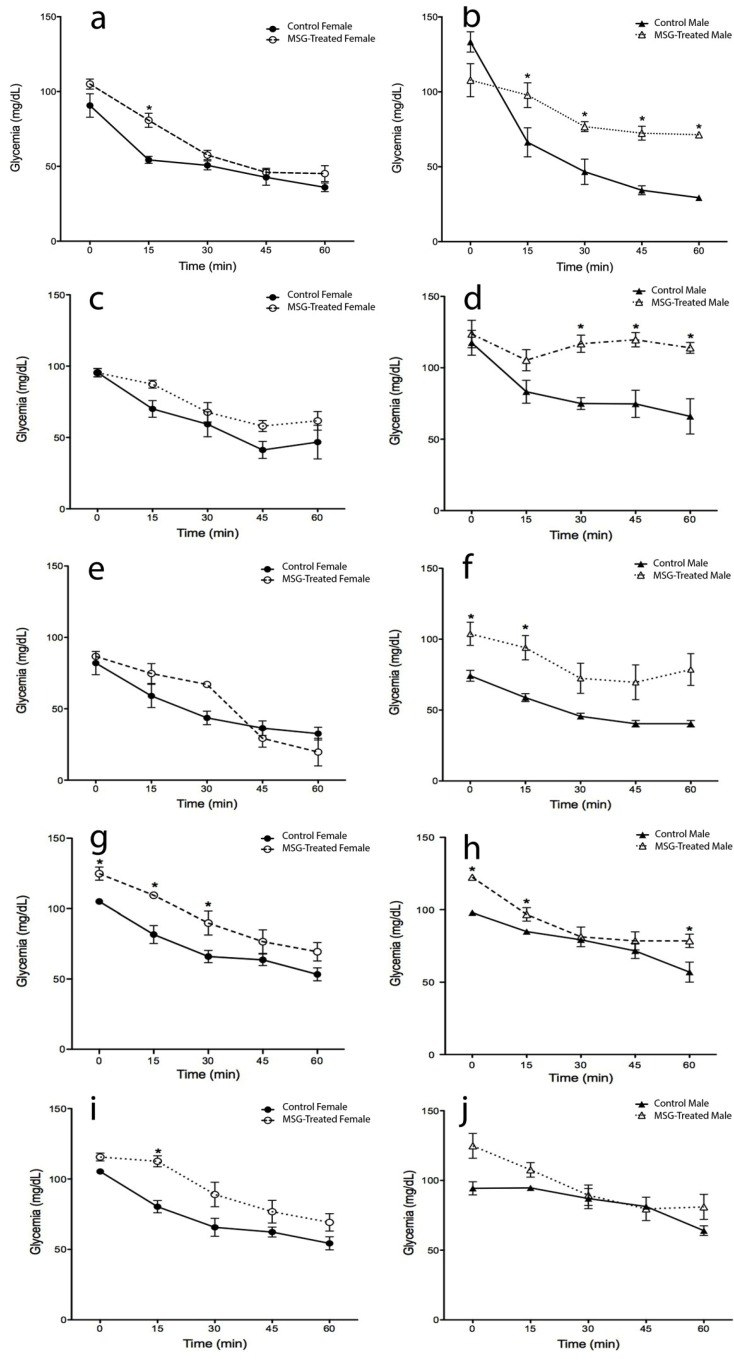
Insulin tolerance test (ITT) in male and female MSG-treated mice*.* ITT was performed by insulin intraperitoneal administration (0.75 IU insulin/kg body wt). Plasma glucose was monitored before (0 min) and 15, 30, 45 and 60 min after insulin-administration, as described in the Materials and Methods section. The determinations were measured at 4 (**a**,**b**), 8 (**c**,**d**), 12 (**e**,**f**), 16 (**g**,**h**) and 20 (**i**,**j**) months of age. Plotted values are the mean ± SE for five mice per group. * *p* < 0.05 *vs.* control female and male; ●, control female; ○, MSG-treated female; ▲, control male; ∆, MSG-treated male.

### 2.3. Insulin Tolerance Test (ITT)

Insulin sensitivity increased gradually in all four groups during the lifetime (Figure 3). No significant differences in glycemic levels were found between MSG-treated and control female mice from 4 to 12 months of age; however, at 16 and 20 months-old, the obese mice showed a 25% increment. When 20-month-old female mice (obese and control) were compared to young female mice (4 months-old), a significant rise in glycemic values (35% and 15%, respectively) was observed (*p* < 0.05). (Figure 3a,c,e,g,i) (*p* < 0.05).

Conversely, obese male mice at four months of age showed 37% higher glycemic values in the first 30 min than the control group. Importantly, statistically higher insulin sensitivity impairments were observed in obese mice during their whole lifespan (8, 12, 16 and 20 months-old). (Figure 3b,d,f,h,j) (*p* < 0.05). 

When insulin sensitivity was compared between genders, MSG-male mice showed 30% lower insulin sensitivity compared with the MSG-female group at four months of age. However, no differences were observed in the following ages. In the control groups, no differences between genders were found, except at 20 months-old, when the males showed 25% higher ITT values at 30 min (*p* < 0.05).

### 2.4. Biochemical Parameters

**Figure 4:**
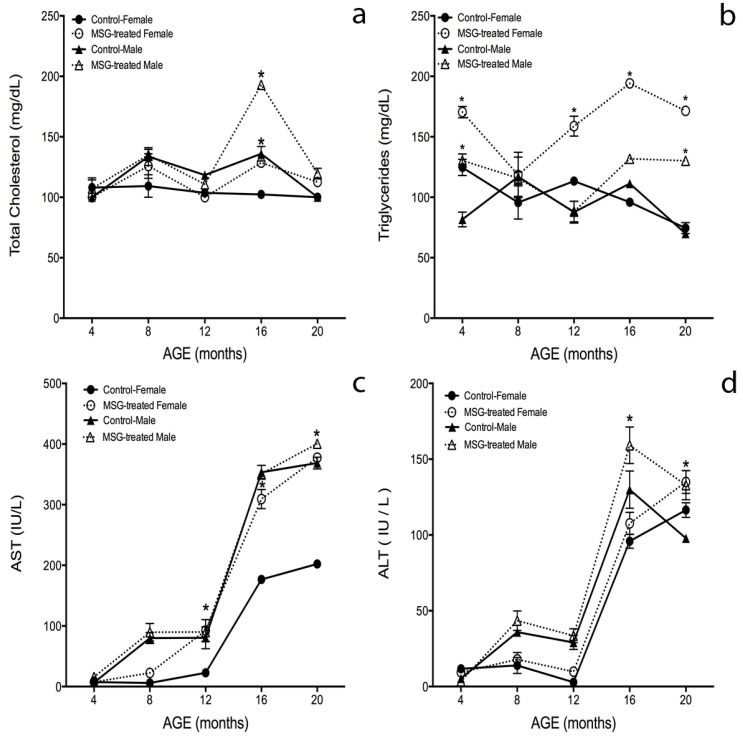
Biochemical parameters in male and female MSG-treated mice. Biochemical parameters: (**a**) total cholesterol; (**b**) triglycerides; (**c**) aminotransferase (AST) and (**d**) alanine aminotransferase (ALT), were determined as described in the Materials and Methods section at 4, 8, 12, 16 and 20 months of age. Plotted values are the mean ± S.E.M. for five mice per group. * *p* < 0.05 *vs.* ●, control female; ○, MSG-treated female; ▲, control male; ∆, MSG-treated male.

Figure 4a shows total cholesterol plasma levels in control and MSG-treated mice over time. The obese mice showed higher cholesterol levels than the control groups from 4 to 20 months-old. Moreover, the cholesterol content was higher in male groups than in female groups. Obese male and female mice increased by 41% and 26% in their cholesterol levels when compared to their control groups at 16 months-old; therefore, MSG-male mice showed 50% more cholesterol than MSG-female mice (*p* < 0.05).

According to Figure 4b, MSG-treated female mice had increased TG levels of 36%, 40%, 100% and 130% at 4, 12, 16 and 20 months-old, respectively, compared to the control group (*p* < 0.05). The only differences observed in TG content between obese males and controls was found at 4 and 20 months, where TG content increased 50% and 55%, respectively, in treated mice. 

No differences were found between genders in control mice; however, triglycerides levels in MSG-treated female were 47% and 31% higher than MSG-treated male at 16 and 20 months-old.

Aminotransferase (AST) levels (Figure 4c) gradually increased along the lifespan, and three of the four groups behaved similarly along the study; only the control females showed a lower AST content (180%) at 16 and 20 months-old compared to the other groups (*p* < 0.05).

Alanine aminotransferase (ALT) determinations (Figure 4d) showed a similar behavior in all groups from 4 to 20 months-old. The male groups presented 30% higher ALT levels than female mice from 8 to 16 months-old. At 20 months, both groups decreased 25% in AST content, but the female control mice increased 12% over the male control group.

When comparing between genders, control males showed higher ALT levels than control females at 8, 12 and 16 months of age, even though, these differences were not significant. Male MSG-treated mice showed a 22% and 35% increment at 16 and 20 months-old, respectively, compared to the control groups. No differences were found between obese female mice and the control group over time (Figure 4d) (*p* < 0.05).

### 2.5. Serum Cytokines Levels

**Figure 5:**
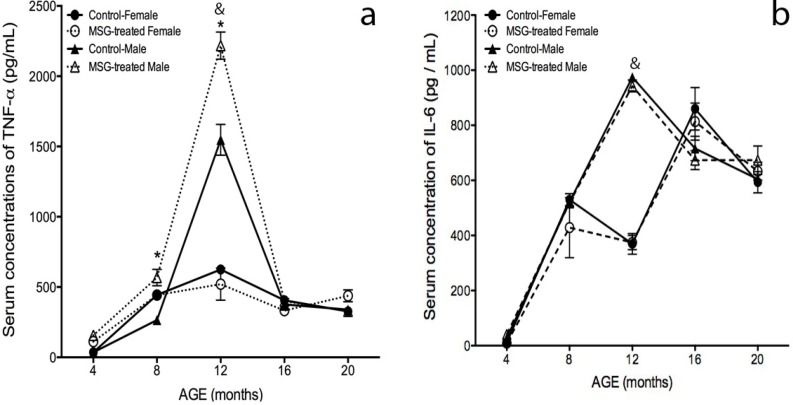
Inflammatory profile in male and female MSG-treated mice. (**a**) TNF-α and (**b**) IL-6 serum levels were quantified by ELISA, as described in the Materials and Methods section at 4, 8, 12, 16 and 20 months of age. Plotted values were the mean ± S.E.M. for five mice per group. * *p* < 0.05 *vs.* control; & *p* < 0.05 MSG-treated female *vs.* MSG-treated male; ●, control female; ○, MSG-treated female; ▲, control male; ∆, MSG-treated male.

To study the inflammatory profile, TNF-α, IL-6 and adiponectin were measured (Figure 5). TNF-α levels gradually increased in the four groups from four months of age, reaching higher values at 12 months-old. These values decreased during the following months. No differences between obese female mice and their control group were found. However, MSG-treated males showed a 43% and 114% increase against their control group at 8 and 12 months-old (Figure 5a).

At 12 months-old, the obese and control male mice showed a 197% and 140% rise in TNF-α levels compared to obese and control female mice groups. At 16 months-old, TNF-α levels decreased in all four groups and remained constant until 20 months of age. 

Figure 5b shows IL-6 measurements. At 12 months-old, the IL-6 concentration in the male groups showed a 163% peak compared to the obese female mice (*p* < 0.05). Four months later, IL-6 levels decreased, and no differences were found between genders.

**Figure 6:**
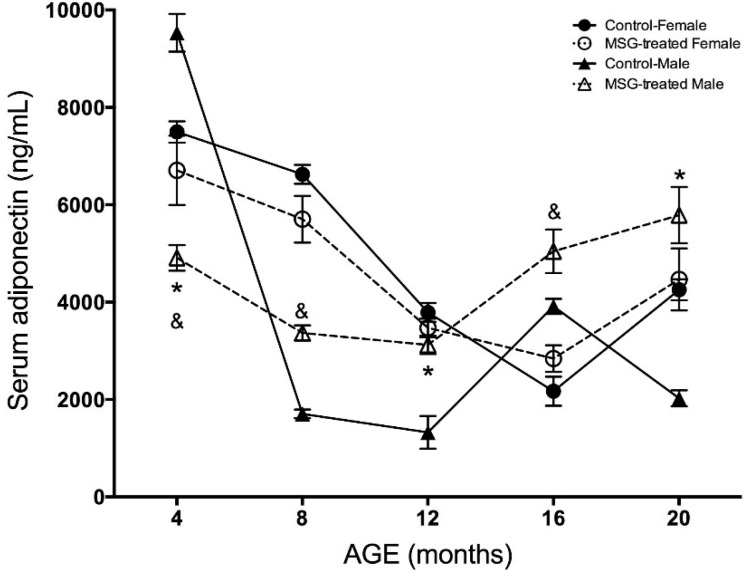
Adiponectin levels (ng/mL) in male and female MSG-treated mice*.* Serum levels of adiponectin were quantified by the ELISA method at 4, 8, 12, 16 and 20 months of age. Plotted values were the mean ± S.E.M. for five mice per group. * *p* < 0.05 *vs.* control; & *p* < 0.05 MSG-treated female *vs.* MSG-treated male; ●, control female; ○, MSG-treated female; ▲, control male; ∆, MSG-treated male.

According to Figure 6, adiponectin concentrations showed a different behavior than TNF and IL-6. Adiponectin decreased along the study in both female and male groups. At 4 months-old, the obese female and male mice showed a 33% and 49%, respectively, drop in adiponectin concentrations compared to their control groups. At 8 and 12 months-old, the hormone diminished in all of the groups, but from 16 months-old, adiponectin levels showed a significant elevation in the obese female and male mice, as well as in the female control group. No differences were found between female groups at the end of the study, but the control males showed 185% less adiponectin concentration compared to obese male mice at 20 months-old. 

## 3. Discussion

The world’s elderly population is growing, and alarmingly, the prevalence of obesity and being overweight has also been estimated to increase linearly with age. Even though there are several reports studying the relationship between obesity and age, there are almost no longitudinal studies to analyze obesity’s effect throughout the whole life, *i.e.*, from the young to the elderly. Therefore, our interest was to evaluate alterations in biochemical and inflammatory parameters, as well as insulin and glucose homeostasis during the obesity-aging process.

We used the obesity MSG-model to generate female and male mice that developed higher body weights than their respective littermates. This weight gain began to decrease at 16 months of age in the four groups, with an inverse correlation at the naso-anal length. Considering the Lee index, the MSG-treated female mice showed the higher obesity index of all of the groups; this is consistent with the studies performed in humans, where women have higher body fat percentages compared to men [15]. Hence, the observed changes might be explained due to an apparent sexual dimorphism, with differences in adipose tissue distribution and abundance. In fact, both female mice groups presented a high subcutaneous fat content, which has been associated with the reduction of ovarian hormone production [16]; meanwhile, in male mice, the adipose tissue predominated in the visceral region [7]. Body weight decline in control and obese mice from 16 to 20 months-old is also consistent with studies in humans, mice and rats, where the changes in adipose tissue mass and function along the lifespan reflect this situation. The weight increase associated with aging is mostly due to fat accumulation in several body depots. It has been reported that in humans, average body weight begins to decay at the age of 60, and by the age of 80, obesity prevalence has decreased by about a third. The weight loss is also a health problem, because it has been associated with sarcopenia, as a result of endocrinology changes and decreased physical activity during aging [17]. The precise reasons responsible for body weight decline with age are complex and still not understood. Fat redistribution during aging occurs in diverse species, and it has been associated with age-related diseases, lipo-toxicity and reduced longevity, but a high subcutaneous to visceral fat ratio has been related with enhanced longevity [15,16,18,19].

It is known that being obese since childhood and throughout life can promote alterations, like diabetes [5,20,21,22,23]; however, it is not clear how fat dysfunction and obesity disturb the aging process. Here, we found an increased glucose intolerance and insulin resistance in MSG-treated mice compared to the control groups, from 4 to 16 months old, which correlated with the body weight gain period. These results agree with previous studies using animals up to 54-week of age [15,24] and has been related to increased adiposity, oxidative stress and inflammation [20,25]. The control male and female mice showed impaired glucose tolerance and insulin resistance throughout the study, but never exceeded the values determined for the obese mice. MSG-treated male and female mice from 4 to 16 months-old showed glucose and insulin tests significantly higher than controls, but after 16 months, the values decreased in both tests in a similar manner as the control groups. This behavior in older obese mice could be correlated with the weight loss and the fat distribution as part of the aging process, in an attempt to reduce risk factors, like inflammation and oxidative stress, which are related to altered glucose homeostasis and insulin secretion. Although little is known about adaptation mechanisms to chronic low-grade stress, like obesity during aging, these adaptive responses must certainly be present to allow the organism to adjust to metabolic alterations. One of several mechanisms in which the insulin sensitivity is lost in obesity is due to the increase production of nitric oxide (NO), particularly as a result of inducible nitric oxide synthase (iNOS), which has also been associated with resistance to insulin [26]. During aging, many factors are known to contribute to insulin sensitivity decrease, such as insulin secretion loss stimulated by glucose and mediated by Sirt1, a β cell sensitivity decrease, mitochondrial function decline, senescent cell accumulation in adipose tissue and increasing oxidative stress. However, the fundamental molecular mechanism of these changes is not clear and is commonly attributed to multiple causes [23,27,28]. 

Our results show a great impairment in male mice glucose tolerance and insulin sensitivity, which could be related to adipose tissue augmentation, since this feature is known to play a significant role in glucose homeostasis and insulin sensitivity control. Moreover, Macotela and collaborators (2009) found an important role for sex steroids in modulating adipose mass and insulin sensitivity. For example, female adipocytes have magnified lipid synthesis compared with male adipocytes in both the perigonadal (PG) and subcutaneous (SC) depots [29]. In females, PG adipocytes also have higher insulin sensitivity to lipogenesis and to insulin signaling. This situation contributes to whole-body insulin sensitivity, allowing female mice to remain insulin sensitive, despite their higher fat mass. Gender differences in visceral adipose tissue diminish at older ages, since postmenopausal females have enlarged visceral fat accumulation [30,31], emphasizing gonadal steroids’ role in this phenomenon. Despite the higher Lee index determined for female mice, it is known that female humans and rodents are more insulin sensitive than males. Thus, women have improved glucose tolerance and increased insulin sensitivity compared to men [32]. In different rodent models of glucose intolerance, insulin resistance and diabetes, males showed a stronger phenotype than females [33,34]. These sex-related differences in insulin sensitivity and adipose tissue development and function could be partially attributable to estrogen and testosterone effects. However, how insulin affects males and females differently and how these differences account for sex-specific adipose tissue development and function regulations are mostly unknown. Diverse structural or anatomical variations are most likely involved in some of the functional differences between adipose tissues from males and females. For example, adipose tissue is innervated by sympathetic efferents, and these innervations are distinct between genders. Interestingly, male rats have more neurons projecting to abdominal fat, whereas females have more projections to subcutaneous fat [35]. The triglyceride levels in female mice were higher than male mice during the all of the experimental time points measured, but MSG-treated animals showed higher TG values than the controls. This result might be explained by dyslipidemia and lipogenesis induction during aging [36,37], along with increments in total lipid levels, free fatty acids, triglycerides and phospholipids [38,39,40,41]. One of the important contributions of our study is the finding that TG seems to be exacerbated by obesity during aging.

As expected, cholesterol levels were higher in obese mice than in control groups; however, when the obese mice were compared by gender, the only statistical difference was observed at 16 months of age, with a significant increase in males compared to females. Therefore, our results agree with previous studies of severe dyslipidemia in MSG-treated female and male mice, characterized by hyper-insulinemia and hyper-cholesterolemia at 17 [42] and 29 weeks of age [12]. No reference studies exist where TG and total cholesterol content were determined in a longitudinal way during obese mice’s lifespan. Hence, another finding of our study is that TG and cholesterol levels at 16 and 20 months-old decreased or were constant in obese mice, coinciding with the changes in glucose and insulin homeostasis at these ages. It is important to mention that aging’s effect on cholesterol homeostasis is poorly defined. A number of epidemiological studies suggest that serum cholesterol levels tend to increase in adults, but subsequently decrease in the very elderly [43]. Similarly, it has been found that obese young and older patients showed significantly higher cholesterol and TG plasma levels compared with their control age matched groups [44]. Interestingly, Karaouzene’s group (2010) showed that younger obese men had relatively larger and accentuated changes in plasma lipids and lipoproteins than the older patients [45]. This result could explain the elevated cholesterol peak found in obese male mice at 16 months of age and the further reduction at 20 months (Figure 4a). Despite this particularly high value, the plasma cholesterol increase occurs continuously throughout life.

There are other studies performed in middle-aged humans, where total cholesterol increased in serum several years before it started to decrease by 0.04 mmol/L a year [46]. Moreover, Abbot and collaborators (1997) found that total cholesterol declined by 1.6–1.8 mg/dL per year with over a 20-year period in elderly men [47]. This value is similar to the decrement in cholesterol observed in healthy young men and women, where the weight loss was the most important factor associated with lipid change [48]. The rise in total cholesterol and its fall in the elderly are significantly associated with similar trends for obesity. A reduction in plasma total cholesterol was highly associated with greater age and a reduction in body mass index over the study interval [49]. These results suggest that the total cholesterol level decline with advancing age may be part of the natural aging process.

Another whole issue is the redistribution of fat outside the fat depots during aging; this condition promotes abnormalities, like non-alcoholic fatty liver disease (NAFLD) [20]. The high body mass index and the large waist circumference observed in obesity are associated with aspartate aminotransferase (AST) and alanine aminotransferase (ALT) elevated levels [50], both of them used as hepatocellular damage markers [51]. Although ALT and AST are found in the liver, they are also present in serum and in various tissues. In particular, ALT serum levels become elevated during liver diseases, and therefore, it is considered a more specific marker for liver injury than AST [52]. When liver function was assessed, we found a gradual increment in ALT and AST levels during the lifespan in obese and control groups and a high AST/ALT ratio, which is used as a reference for liver damage. Interestingly, the male groups had superior ALT values compared to female groups over time, this difference decreasing at 20 months of age. Furthermore, AST in the female control group was lower compared to the other three groups. To our knowledge, there are almost no studies assessing liver markers in obese mice during aging; however, studies in humans have shown that AST is slightly increased in obese males; while in females, the ALT and AST levels did not change with obesity, and the levels of these enzymes were well below the levels reported in males [28,53]. Similarly, Choi and coworkers studied human obesity and found that the male cohort displayed higher AST and ALT values, than obese women [54]. In our model, ALT levels decreased in male mice from 16 to 20 months-old; however, obese mice’s ALT values remained higher relative to their control. In female mice during the same time period, ALT levels were also augmented, but not with the same intensity compared to the previous months. These results agree with the ones by Dong and collaborators, where ALT levels decreased with age in both men and women, independent of metabolic syndrome components, adiposity signaling biomarkers and other commonly used liver function tests [55]. Further studies are needed to understand the mechanisms responsible for the ALT decline with age; however, it seems clear that a different cutoff value in ALT and AST between genders in the elderly should be considered. 

Adipose tissue mass accumulation in the visceral region during obesity has been associated with elevated levels of inflammatory mediators, including serum C-reactive protein (CRP), acute phase proteins and pro-inflammatory cytokines TNF-α and IL-6 [56,57,58]. In general, inflammation plays an important role in insulin resistance, diabetes mellitus type 2 and cardiovascular risk progression in the elderly [56,59]. Hence, the low-grade systemic inflammation characterized by raised CRP and pro-inflammatory cytokines during aging has been termed inflammaging and has been linked to increased oxidative stress. In agreement with that, in this study, the pro-inflammatory serum markers, TNF-α and IL-6, increased in MSG-treated and control mice over time. The cytokine rise correlated with obesity, the Lee index and the glucose and insulin homeostasis results. This correlation is consistent with the fact that TNF-α is a key factor during insulin sensitivity loss pathogenesis [58,60,61,62]. Male mice showed higher TNF-α expression compared to female mice along the study, but MSG-treated male mice had even higher TNF-α levels compared to control mice. No differences were found between the female obese and control groups. Serum IL-6 levels gradually increased over time in the four groups, and at 12 months of age, a significant increment in male mice compared to female mice was observed. No differences between MSG-treated mice and their controls were found. It is important to note that pro-inflammatory cytokines levels decreased in all groups from 12 to 20 months-old, without differences between them. To explain the TNF-α effect on insulin sensitivity, three possible mechanisms have been proposed: (1) through abnormal insulin receptor substrate (IRS)-1 phosphorylation; (2) by glucose transporter 4 (GLUT-4) loss in the adipocyte; and (3) by adiponectin suppression [56,57,63]. 

It is important to say adiponectin’s precise role in diabetes is not well characterized, although it is considered a beneficial adipokine, showing negative correlations with many age- and obesity-related diseases and a positive correlation with longevity and insulin sensitivity [64]. Previous results from our group showed that lower adiponectin concentrations in obese animals were associated with chronic inflammation, insulin resistance and diabetes mellitus type 2 [65]. Here, adiponectin levels decreased gradually from 4 to 12 months-old, with significant differences between MSG-treated female and male mice. In this time period, the mice showed body weight gain and elevated TNF-α levels, as well as an altered glucose and insulin homeostasis. However, from 16 months-old-on, adiponectin levels improved in obese and control mice, while body weight and TNF-α reduced. Previous studies have reported that adiponectin concentrations increase with age in humans [66]. Circulating adiponectin levels positively correlate with insulin sensitivity induced by the nuclear receptor PPAR-γ activity in humans and rodents [67,68,69]. Comparing by gender, male mice showed lower adiponectin levels in comparison to female mice. A sexual dimorphism in adiponectin circulating levels has been shown in mice, since females present higher levels than males [70,71]. This fact was also corroborated in our study; however, the direct influence of androgens on adiponectin concentrations is not clear yet. It has been suggested that sexual hormones might regulate adiponectin production, although it is still controversial how this regulation is being performed during the organismal lifetime. This might partly explain why females are more susceptible to insulin than males [72], because adiponectin concentrations negatively correlate with fat visceral mass, but positively with subcutaneous fat mass [73]. In the MSG-treated mice model, we found that obesity induced a low-grade chronic inflammatory state accompanied by pro-inflammatory cytokine (TNF-α and IL-6) increments, with adiponectin gene expression reduction [74] and plasma adiponectin elevation following weight loss. Still, more detailed analyses of the association among adiponectin, gender, aging and metabolic risk factors are necessary.

One of the main findings of our work was the damage attenuation or adaptation effect associated with increased age, both in control and MSG-treated mice. At the beginning of the study, female and male obese mice showed increased metabolic alterations. However, at middle and older ages, when all of the groups started losing weight, they improved the glucose tolerance and insulin action. This effect was stronger in MSG-treated mice, diminishing in this way the differences in comparison to the control groups. Another factor that possibly contributed to this attenuation is the adiponectin increase during aging, which positively correlates with weight loss and insulin sensitivity improvement. It is known that weight reduction in obese elderly people improves the cardiovascular risk profile, reduces chronic inflammation and correlates with a better life quality [75]. 

Our results show that obesity effects were attenuated during aging, despite the alterations first observed at young ages. Increasing evidence suggests that the association between obesity and mortality declines with advancing age in both genders [76]. Therefore, it might be important to discuss the paradigm that obesity is always associated with a significantly higher risk of all-cause mortality [77], because it is unknown if the obesity-mortality association is sustained at old age. Wang recently reported a systematic meta-analysis on the obesity-mortality association in men and women; his results support the weakening trend of the obesity-mortality association with increasing age. Therefore, obesity may play a more important role in the elevated mortality risk in younger people than in older people [78].

## 4. Materials and Methods

### 4.1. Chemicals

All chemicals and reagents were purchased from Sigma Chemical Co. (St. Louis, MO, USA). The reagents obtained from other sources are detailed throughout the text.

### 4.2. Animals

CD-1 mice (*Mus musculus*) were obtained from the closed breeding colony at the Universidad Autonoma Metropolitana Iztapalapa (UAM-I). Mice were handled according to international and national ethical standards, taking into account the Official Mexican Rule (NOM-062-ZOO-1999, revised in 2001) and the International Guide for Laboratory Animals Caring and Use NRC 2002. The experimental protocol was approved by the University Ethics Committee for Animal Experimentation (UAM, CDCBS.127.08). 

### 4.3. Obesity Induction with Monosodium Glutamate (MSG-Treated Mice)

MSG-induced obesity was carried out in CD-1 mice by neonatal neuro-intoxication with monosodium glutamate (MSG), as described before [6]. On the day of birth, pups were randomly divided into two groups upon delivery. On postnatal Days 2 and 4, MSG-treated pups were injected subcutaneously (SC) with MSG (2 mg/kg body weight dissolved in 0.01 mL/kg saline solution), and further, 4 mg/kg injections were performed on Days 6, 8 and 10. Control pups were SC injected with equivalent volumes of isotonic saline solution [14]. After weaning, MSG and control mice were again separated by gender. Biochemical and physiological determinations were performed in the four mice groups at 4, 8, 12, 16 and 20 months of age. Animals were given standard commercial diet (Harlan 2018S, Harlan Teklad, Madison, WI, USA), water *ad libitum* and were housed under a controlled environment room (55% humidity, 21 ± 1 °C, 12:12 h light-dark cycle).

### 4.4. Lee Index (LI) Quantification

Lee index is usually used to quantify the mice obesity index by dividing the body weight cubic root (g), by the nose-to-anus length (cm) [79]. Mice length was measured with calipers, and body weight (BW) was determined using a sensitive electronic balance. 

### 4.5. Oral Glucose Tolerance Test

The oral glucose tolerance test (OGTT) was performed at 4, 8, 12, 16 and 20 months of age. Overnight (12 h) fasted mice were orally administered with dextrose anhydrous (2 g/kg body wt) by gavage. Blood samples were obtained from the tail vein at 0, 30, 60, 90, 120 and 150 min after dextrose administration. Blood glucose levels were determined with the glucose dehydrogenase method (Roche Diagnostics, Mannheim, Germany) [8].

### 4.6. Insulin Tolerance Test

Insulin tolerance test (ITT) was performed at 4, 8, 12, 16 and 20 months of age. Overnight fasted animals were intra-peritoneal injected with insulin (0.75 IU/kg body wt). Blood samples (20 μL) were collected from the tail vein. Measurements were performed at 0, 15, 30, 45 and 60 min, and blood glucose levels were measured in the same way as in the OGTT [8,80].

### 4.7. Sera Samples Preparation

Mice were sacrificed at 4, 8, 12, 16 and 20 months of age. The blood samples were allowed to clot at room temperature for 1 h and centrifuged at 2000× *g* for 15 min. Sera samples were stored at −80 °C until assayed. The serum was used for biochemical parameter measurement and inflammatory profile determinations.

#### Biochemical Parameters

Alanine aminotransferase (ALT) and aspartate aminotransferase (AST), as well as triglycerides and total cholesterol were determined spectrophotometrically from serum using a Reflotron System (Roche Diagnostics, Indianapolis, IN, USA) [8].

### 4.8. Enzyme-Linked Immunosorbent Assays (ELISA)

Serum cytokines, IL-6, TNF-α (Thermo Fisher Scientific, Rockford, IL, USA) and adiponectin (Invitrogen of Life Technologies Corporation, Frederick, MD, USA) expression were assayed by an enzyme-linked immunosorbent assay (ELISA) [8].

### 4.9. Statistical Analysis

Values are expressed as the mean ± SEM (*n* = 5 in each group). Statistical analyses were performed by a one-way ANOVA followed by Tukey’s *post hoc* multiple comparison test. All *p*-values < 0.05 were considered statistically significant. All statistical analyses were done using the NCSS 2000 software package (NCSS, Kaysville, UT, USA).

## 5. Conclusions

Our data agree with previous reports where alterations in metabolic control and increased inflammation have been observed in obese female and male mice. However, our data suggest the existence of an attenuation or adaptation effect at older ages, which might weaken the association between obesity and mortality with advanced age in both genders. Future research is necessary to understand the processes that relate inflammation and oxidative stress with obesity during aging, as well as to unveil the adaptation mechanisms, which might induce protective cellular responses. 
